# Insomnia, negative affect, and psychotic experiences: Modelling pathways over time in a clinical observational study

**DOI:** 10.1016/j.psychres.2018.08.090

**Published:** 2018-11

**Authors:** Sarah Reeve, Alecia Nickless, Bryony Sheaves, Daniel Freeman

**Affiliations:** a Department of Psychiatry, University of Oxford, Warneford Hospital, Oxford, UK; b Nuffield Department of Primary Care Health Sciences, University of Oxford, Radcliffe Observatory Quarter, Oxford, UK; c Oxford Health NHS Foundation Trust, Warneford Hospital, Oxford, UK

**Keywords:** Paranoia, Hallucinations, Schizophrenia, Depression, Anxiety, Longitudinal, Mediation

## Abstract

Insomnia has been shown to contribute to the development of psychotic experiences, predominantly via increasing negative affect. However, the role of insomnia in the persistence of psychotic experiences is yet to be investigated in a clinical population. Furthermore, other plausible influences, such as psychotic experiences contributing to insomnia, remain to be evaluated. This study tests the role of insomnia as a predictor of persistence of psychotic experiences versus other potential causal routes. Twenty-nine patients aged 18–30 with non-affective psychosis completed three assessments over three months of their insomnia, negative affect, and psychotic experiences. Mixed effect models allowed comparisons between hypothesis-based models (comprising insomnia as predictor, negative affect as mediator, and psychotic experiences as outcome) and oppositional models, where relationships were reversed. The results supported the hypothesised mediation model above models where negative affect was primary. Insomnia was also found to be a stronger predictor of later hallucinations than vice versa, although a bidirectional relationship was indicated between insomnia and paranoia. In conclusion, insomnia predicts persistence of psychotic experiences over time to the same or greater extent than psychotic experiences contribute to insomnia. This supports insomnia as a potential intervention target in psychosis.

## Highlights

• Insomnia, negative affect, and psychotic experiences are all related across time.
• Negative affect mediates the insomnia and psychotic experience relationship.
• Insomnia predicts later hallucinations to a greater extent than vice versa.
• Insomnia and paranoia have a bidirectional relationship across time.

## 1 Introduction

Insomnia has traditionally been thought of as a consequence of psychotic symptoms, however recent research indicates that insomnia itself contributes to the development of psychotic experiences (Reeve et al., 2015). For example, an experimental study found that inducing insomnia-like sleep loss in non-clinical volunteers resulted in increased paranoia and hallucinations (Reeve et al., 2018), and a large clinical trial of an online CBT intervention for insomnia in students found that treating insomnia reduced subclinical paranoia and hallucinations (Freeman et al., 2017). Together these findings demonstrate a causal role for insomnia in psychosis, implying that insomnia may represent a novel target for treatment of psychosis.

Sleep disturbance in general has been increasingly associated with psychotic experiences in both clinical and non-clinical populations (Chiu et al., 2016, Davies et al., 2017, Koyanagi and Stickley, 2015) . However, there is a surprising lack of studies investigating the relationship between insomnia (as a specific sleep disorder) and psychosis in individuals with a psychotic disorder (Reeve et al., 2015). Cross-sectional studies indicate that individuals with psychotic disorders and comorbid insomnia have more severe psychotic experiences than those without (Freeman et al., 2009, Xiang et al., 2009). Yet longitudinal research is currently limited to studies utilising experience sampling methods (ESM) to collect high frequency data across a short time period. This technique allows investigation of the interplay between night-time sleep and day-time mental health, with significant relationships reported between lowered sleep quality, efficiency, and duration and increased psychotic experiences the following day (Hennig and Lincoln, 2018, Mulligan et al., 2016, Waters et al., 2011). Notably, in one of these studies shorter sleep was found to predict paranoia (but not the reverse) in a non-clinical adolescent group (Hennig and Lincoln, 2018).

However, there are limitations to the ESM approach. Firstly, although the results are clearly applicable to insomnia (in which sleep efficiency, quality, and duration are lowered), these studies do not measure insomnia symptoms directly. Secondly, as assessments are completed repeatedly within a short time, outcomes are measured using individual items (or a small set of items), instead of a fully validated questionnaire or interview assessment. Finally, due to the intensity of the ESM approach the observational period remains short. Therefore, it remains unclear if the day-by-day relationships found in ESM studies can be extrapolated to diagnosable insomnia (which requires a duration of 3 months of symptoms) or psychotic symptoms assessed over longer time periods. This is of particular importance in early psychosis where treatment of predictors of psychotic symptoms could improve later clinical trajectory.

**Fig. 1:**
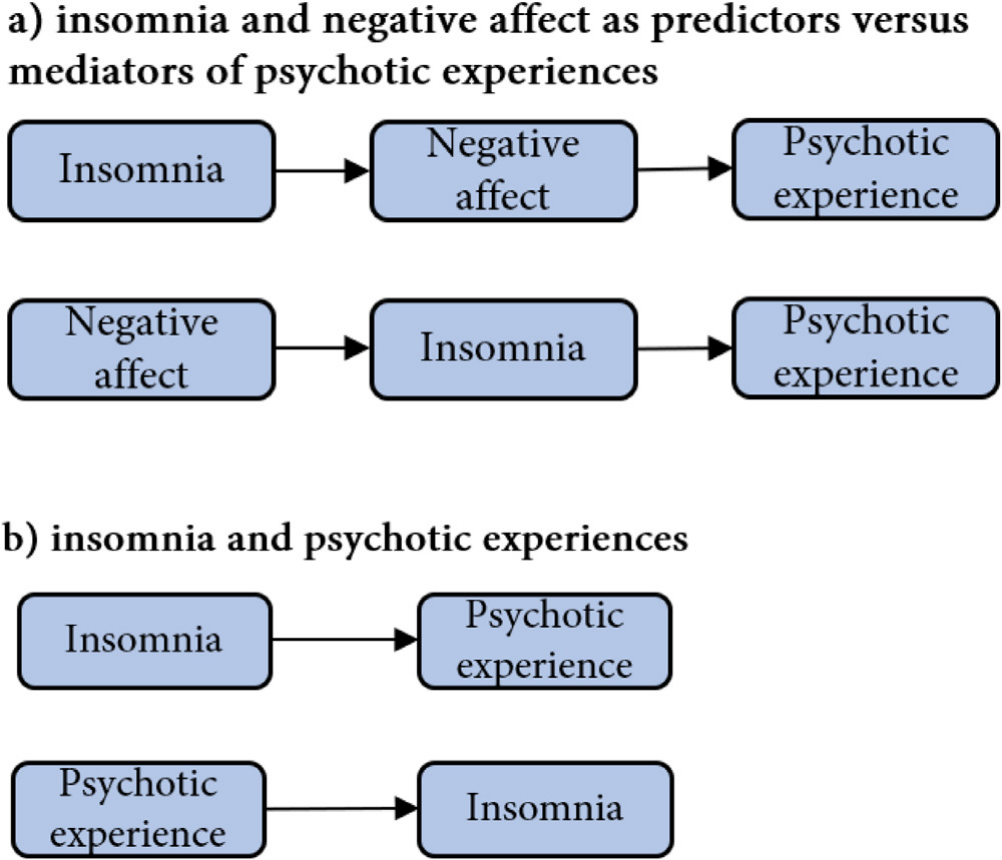
Possible pathways between insomnia, negative affect, and psychotic experiences.

Negative affect – here used as a generic term to refer to depression and anxiety - is often identified as a mediator in the insomnia to psychosis relationship (e.g. Reeve et al., 2018, Reeve et al., 2015), as supported by a large literature linking insomnia and affect. Insomnia and depression are strongly related: individuals with insomnia are at higher risk of developing depression (Li et al., 2016), and treating insomnia has also been shown to improve depression (e.g. Christensen et al., 2016). Those with depression are also more likely to develop insomnia (Jansson-Fröjmark and Lindblom, 2008). Insomnia and anxiety have a strong, if less researched relationship. Anxiety is predictive of later insomnia, and insomnia is similarly predictive of later anxiety (Neckelmann et al., 2007). The psychological processes shared between cognitive models of insomnia and anxiety (such as hyperarousal, catastrophising, and intrusive thoughts) also link the phenomena (Espie, 1991, Harvey, 2002). Whilst negative affect has been shown to mediate the relationship between insomnia and psychosis (Hennig and Lincoln, 2018, Mulligan et al., 2016, Reeve et al., 2018), the bidirectional relationship between insomnia and negative affect means that it is equally plausible that insomnia could mediate the relationship between negative affect and psychosis (see Fig. 1a for a diagram of these pathways). No previous study has tested this possibility.

Furthermore, while recent research has focused on demonstrating the causal role of insomnia in psychotic experiences (Freeman et al., 2017, Reeve et al., 2018), it also remains likely that psychotic experiences contribute to insomnia (see Fig. 1b). One obvious route would be that distress from psychotic experiences increases arousal and delays sleep onset (Waite et al., 2016a). Other possible factors include lowered daytime activity, which is common in psychosis (Hodgekins et al., 2015, Stubbs et al., 2016), and can cause sleep disturbance by destabilising circadian rhythms (Waite et al., 2016b). Based on these and other factors a bidirectional relationship between insomnia and psychosis has been proposed, but not adequately tested (Harvey and Murray, 2011, Reeve et al., 2015). Whether this relationship is truly bidirectional is important to assess, since it may have clinical implications for prioritising treatment of insomnia versus psychotic experiences.

### 1.1 The current study

The current study aimed to investigate the interaction between insomnia, negative affect (i.e. depression and anxiety), and psychotic experiences (i.e. paranoia and hallucinations) over several months within a cohort of individuals with early psychosis. The analytical approach was to test the directions of effect between insomnia, negative affect, and psychotic experiences by comparing models derived from key hypotheses (that insomnia predicts later psychotic experiences, with negative affect acting as the key mediator) to oppositional models where key relationships are reversed in order to disentangle the most relevant causal influences between these factors. The hypotheses tested were:

1. Insomnia, negative affect, and psychotic experiences are cross-sectionally associated;
2. Insomnia is predictive of later psychotic experiences;
3. The relationship between insomnia and later psychotic experiences is mediated by negative affect;
4. Psychotic experiences are predictive of later insomnia;
5. Insomnia is more predictive of later psychotic experiences than psychotic experiences are of later insomnia.

## 2 Method

### 2.1 Recruitment

Twenty-nine participants were recruited for the current study. The inclusion criteria were: primary diagnosis of non-affective psychotic disorder; outpatient status; and age between 18 and 30. The age range was selected to minimise the effects of long-term antipsychotic medication usage on sleep, and to control for changes in sleep over the lifespan. Exclusion criteria were: primary diagnosis of affective, substance abuse, organic, or neurological disorder; and non-fluency in English. Eligible participants were initially approached by members of their NHS care team and given information regarding the study. Those willing to participate provided written informed consent to take part in the study and received compensation for their time in taking part. The study received approval from an NHS research ethics committee (South West-Frenchay REC reference 15/SW/0291), and local approvals were received for each of the three study sites.

### 2.2 Design and assessments

In this longitudinal observational study participants were assessed at baseline, one month, and three months. All measures were completed at every time point. For all measures higher scores indicate greater severity of symptomatology.

#### 2.2.1 Insomnia

Insomnia was assessed using the Sleep-50 (Spoormaker et al., 2005), a self-report questionnaire indexing severity of a number of sleep disorders. The total scale is comprised of 50 statements which are rated for agreement over the past month, on a 1 (“Not at all”) to 4 (“Very much”) Likert scale. The subscale for insomnia was used in the current study, which comprises 8 items, with a minimum score of 8 and a maximum score of 32. The insomnia subscale demonstrates high consistency (Cronbach's alpha = 0.85)

#### 2.2.2 Psychotic experiences

Paranoia and hallucinations were assessed using the Specific Psychotic Experiences Questionnaire (SPEQ; Ronald et al., 2014). The SPEQ is a self-report questionnaire with dimensions for individual psychotic experiences, of which the subscales for paranoia and hallucinations were used in the current study. These subscales have high internal consistency (Cronbach's alpha 0.93 for paranoia, 0.87 for hallucinations), and have been used in clinical and non-clinical groups (Ronald et al., 2014; Zavos et al., 2014). The paranoia subscale contains 15 items, and the hallucinations subscale contains 9 items, both assessing the frequencies of each psychotic experience over the past month. Items are rated on a 0–5 Likert scale (where 0 is “Not at all”, and 5 is “Nearly all the time”), therefore the maximum score for paranoia is 75 and the maximum score for hallucinations is 45.

#### 2.2.3 Negative affect

Depression and anxiety were assessed using the relevant two subscales from the 21-item Depression Anxiety Stress scale (DASS; Lovibond and Lovibond, 1995). The DASS is widely used and well-validated in clinical groups, with high internal consistency (Cronbach's alpha 0.94 for depression, 0.87 for anxiety) In this questionnaire there are seven items indexing each of depression and anxiety, all rated on a 0–3 Likert scale where 0 is “Did not apply to me” and 3 is “Applied to me very much or nearly all the time”, therefore the maximum score for depression or anxiety is 21.

### 2.3 Analysis

All analysis was carried out in SPSS 23 (IBM Corp., 2015). The first step in the analysis was to examine descriptive statistics in order to report on the levels of insomnia, paranoia, hallucinations, depression and anxiety, and indicate if there were changes over the course of the three-month observation period.

For all further analyses the study variables were transformed by log_10_. This allows the estimated coefficients and t-statistics to be compared across analyses (Benoit, 2011, Box et al., 1964, Keene, 1995). The coefficient can be interpreted as a percentage contribution, such that if the coefficient of insomnia is 1.7 towards paranoia, this indicates that a 1% increase in insomnia is associated with a 1.7% increase in paranoia. Prior to log transformation 1 was added to all scores to preserve 0 scores in the raw data (log_10_(1) is 0, whereas log_10_(0) is incalculable as it tends to negative infinity).

#### 2.3.1 Phase 1: cross-sectional association

In this phase, cross-sectional associations were tested between insomnia, negative affect (i.e. depression and anxiety) and psychotic experiences (i.e. paranoia and hallucinations), with the hypothesis being that all study variables are significantly associated within each time point. Multi-level mixed effect models were used, allowing these tests to be nested within each individual and within each time point. Fixed effects were assumed in these and all subsequent multi-level mixed effect models, with random effects used to account for repeated measures from the same participant.

#### 2.3.2 Phase 2: longitudinal mediation

**Fig. 2:**
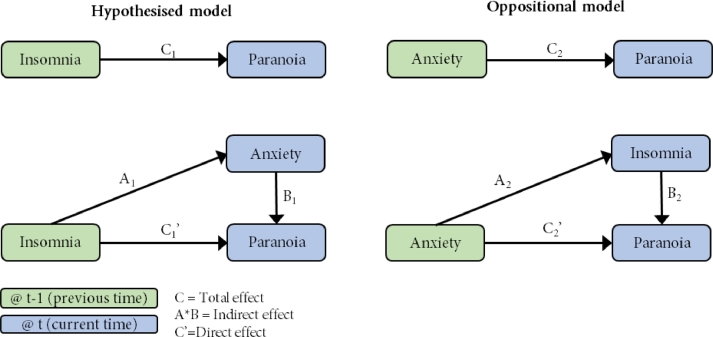
Longitudinal mediation analysis process diagram (phase two).

In this phase the second and third hypotheses were tested by applying a series of mixed effect models to longitudinal data. The second hypothesis was tested by examining the significance of the predictive relationship from insomnia to later psychotic experiences. The third hypothesis was addressed by comparing the fit of a hypothesised model (where insomnia is the predictor, and depression or anxiety was the mediator) to an opposition model (where anxiety or depression was the predictor, and insomnia was the mediator) with psychotic experiences (paranoia or hallucinations) as the outcome variable. See Fig. 2 for an outline of these pathways. In all cases the predictor was taken from the previous time point (t−1), and the mediator and outcome were taken from the current time point (t), amalgamating the three time points into two parallel longitudinal comparisons.

Model fit was compared by using the Bayesian Information Criterion (BIC). This provides a measure of model fit to the observed data, with a penalty function applied for lower parsimony. The BIC value can be compared across different models which are modelling the same outcome, with a lower BIC indicating that the model is a better fit to the observed data. Therefore the hypothesised models were expected to have a lower BIC value than the oppositional models. Comparison between models can be achieved by using threshold values of differences between BICs, with differences greater than 5 and greater than 10 indicating strong or very strong support for a significant difference between the two models (Kass and Raftery, 1995).

Mediation was estimated using the Baron and Kenny method (Baron and Kenny, 1986). In this method the relationship between predictor and outcome (e.g. insomnia and paranoia) is estimated to give the total effect (Path C). The relationship between the predictor and hypothesised mediator (e.g. insomnia to anxiety) is estimated (Path A). Finally, the relationship between predictor and outcome (e.g. insomnia to paranoia) is estimated, with the addition of the mediator (e.g. anxiety) to the model. This gives the estimate for Path B (from the mediator to the outcome) and Path C’ (from predictor to outcome when controlling for the mediator). See Fig. 2 for a diagram of this process. The proportion of mediation is calculated as the ratio of the indirect effect (the product of Path A and Path B) to the total effect. Proportions greater than 100% indicate that the mediator has an independent relationship with the outcome in addition to its role as a mediator in the given model. This proportion was compared in the hypothesised and oppositional models, with the expectation that hypothesised models show a higher proportion of mediation than oppositional models.

#### 2.3.3 Phase 3: directional comparison

Finally, a comparison was made of the predictive value for insomnia to later paranoia or hallucinations versus the predictive value for paranoia or hallucinations to later insomnia in order to address hypotheses four and five.

**Fig. 3:**
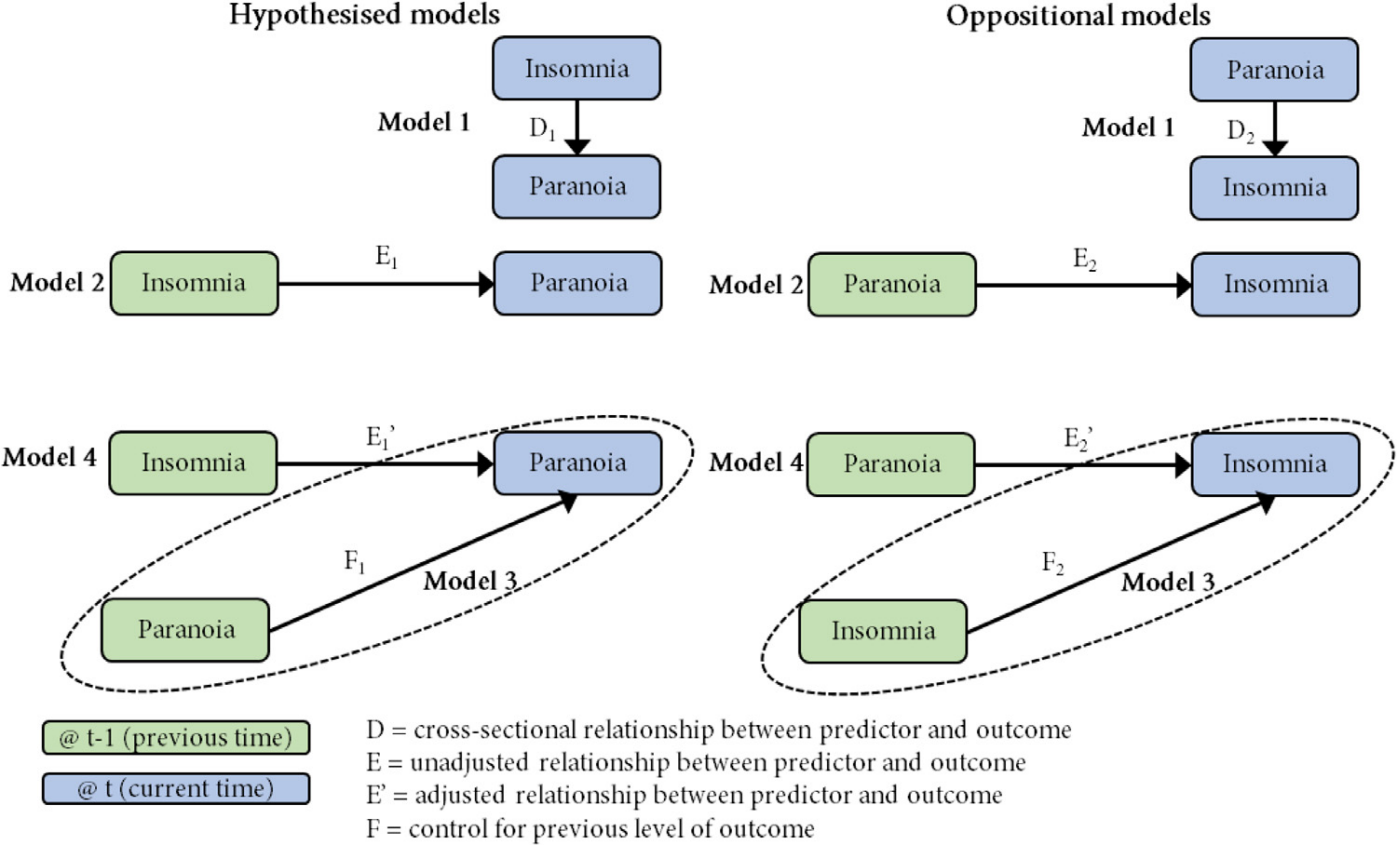
Longitudinal directional analysis process diagram (phase three).

Another set of mixed effects models were fitted, again with hypothesised models compared against oppositional models. A diagram of this analysis process can be found in Fig. 3. First, univariate models were fitted with current insomnia, previous insomnia (Model 2 in the figure), and previous paranoia as factors predicting current paranoia. Following this previous paranoia and previous insomnia were tested as predictors of current paranoia in the same model (Model 4 in the figure). This allows examination of the contribution of previous insomnia to predicting change in paranoia by controlling for the influence of previous paranoia on later paranoia. The oppositional model was then constructed (in this case with paranoia as a predictor of insomnia) to compare to the hypothesised model. The same process was then repeated for insomnia and hallucinations.

Hypothesis four predicted that the psychotic experiences would significantly predict later insomnia, which is tested in the oppositional models at this phase. Hypothesis five was then addressed by comparing the hypothesised and oppositional models. It was not possible to use the BIC for comparison as the outcome variable differed between models, but the *p*-values, *t*-statistics, and estimated coefficients were instead used to compare the strength of the associations in each model.

## 3 Results

### 3.1 Demographic and descriptive statistics

Demographic statistics can be found in Table 1. The study group had a slight majority of female participants (*n *= 16, 55.2%). The majority of participants were patients within an early intervention in psychosis service (*n *= 21, 72.4%), and the majority were prescribed antipsychotic medication *(n *= 21, 72.4%).

**Table 1:** Demographic statistics of study sample.

Demographic	Value
Age - mean (SD)	23.55 (3.8)
Gender – *n* (%)	
Male	13 (44.8)
Female	16 (55.2)
Living status – *n* (%)	
With parents or other relatives	15 (51.7)
Alone	5 (17.2)
With spouse/partner	5 (17.2)
Other (e.g. shared accommodation)	4 (13.8)
Antipsychotic Medication	
Prescribed antipsychotic medication - *n* (%)	21 (72.4)
DDD - mean (SD)^a^	0.81 (0.4)
Mental health team type – *n* (%)	
EIS	21 (72.4)
AMHT	8 (27.6)
NHS Trust – *n* (%)	
OHFT	20 (69.0)
CNWL	8 (27.6)
BHFT	1 (3.4)
Ethnicity/Citizenship – *n* (%)	
White/White British	15 (51.7)
Asian/Asian British	5 (17.2)
Black/African/Caribbean/Black British	3 (10.3)
Mixed or multiple ethnic background	6 (20.7)

AMHT = Adult Mental Health Team; DDD = Defined Daily Dose; EIS = Early Intervention in Psychosis Service; OHFT = Oxford Heath NHS Foundation Trust; CNWL = Central and Northwest London NHS Foundation Trust; BHFT = Berkshire Healthcare NHS Foundation Trust.

a DDD average excludes the eight participants not prescribed antipsychotic medication at time of participation.

Descriptive statistics for the study measures are displayed in Table 2. There was high retention within the study, with 89.1% (*n *= 26) and 96.6% (*n *= 28) of participants completing the one and three-month follow-up assessments. Severity of all symptoms decreased over the observation period, with the largest decreases seen in anxiety, (48.3% reduction from baseline to three months), hallucinations (19.8%), and paranoia (17.6%).

**Table 2:** Descriptive statistics of study measures across time points.

	Baseline (*n *= 29)	1 month (*n *= 26)	3 months (*n *= 28)
	Mean (SD)	Mean (SD)	Mean (SD)
**Insomnia** (Sleep-50)	21.45 (6.3)	20.58 (6.4)	18.68 (6.4)
**Paranoia** (SPEQ)	38.72 (23.5)	34.08 (24.1)	31.89 (24.1)
**Hallucinations** (SPEQ)	18.07 (12.8)	15.58 (11.9)	14.50 (14.0)
**Depression** (DASS)	9.69 (5.5)	9.81 (7.7)	8.75 (7.3)
**Anxiety** (DASS)	12.31 (6.3)	8.50 (5.8)	6.36 (4.4)

SPEQ = Specific Psychotic Experiences Questionnaire; DASS = Depression Anxiety Stress Scale.

### 3.2 Phase one: cross-sectional associations

The results of the cross-sectional analysis can be seen in Table 3. In the univariate models, current insomnia, anxiety, and depression were all significantly positively associated with current paranoia (*p* < 0.001). Current insomnia, anxiety and depression were also significantly positively associated with increased hallucinations (*p* < 0.001). Current insomnia was significantly associated with anxiety and depression (*p* < 0.001). In summary, all the factors (insomnia, anxiety, and depression) were significantly cross-sectionally associated with each other, as predicted by the first hypothesis.

**Table 3:** Associations between insomnia, psychotic experiences, and negative affect (cross-sectional analysis).

Factors	Outcome	BIC	Beta	Std. Error	df	*t*	*p*	95% CI
Insomnia	Paranoia	138.0	2.073	0.41	80.1	5.10	<0.001	1.2, 2.9
Anxiety	Paranoia	100.0	1.154	0.12	77.0	9.84	<0.001	0.9, 1.4
Depression	Paranoia	74.9	1.118	0.09	67.9	12.67	<0.001	0.9, 1.3
Insomnia	Hallucinations	147.3	1.278	0.43	80.0	2.97	0.004	0.4, 2.1
Anxiety	Hallucinations	118.8	0.940	0.13	77.9	7.12	<0.001	0.7, 1.2
Depression	Hallucinations	124.3	0.783	0.12	69.8	6.40	<0.001	0.5, 1.0
Insomnia	Anxiety	39.5	1.787	0.22	77.7	8.14	<0.001	1.3, 2.2
Insomnia	Depression	52.6	1.819	0.23	64.9	7.98	<0.001	1.4, 2.3

### 3.3 Phase two: longitudinal mediation analysis

Table 4 contains results from the longitudinal analysis, investigating the hypothesised ‘insomnia to negative affect to paranoia’ pathway, compared with an oppositional ‘negative affect to insomnia to paranoia’ pathway. All total effect pathways were significant (i.e. previous insomnia, anxiety and depression were all significantly associated with later paranoia). The indirect pathway section shows that, while all relationships were highly significant, the estimated coefficients for the hypothesised models were higher than in the oppositional models. For example, a 1% increase in insomnia resulted in a 2% increase in later depression, whereas a 1% increase in depression resulted in a 0.2% increase in later insomnia. The final section of the table illustrates the competing possibilities for mediation. The BIC values show that the hypothesised models where insomnia is prior were a better fit to the data (BICs of 66.8 and 45.6) than models where anxiety or depression are prior (BICs of 86.1 and 83.6), with the size of the differences (both > 10) provide ‘very strong’ support for a significant difference between the models in both cases (Kass and Raftery, 1995). The mediated portion of the effect was higher in the hypothesised models than in the oppositional models.

**Table 4:** Insomnia, anxiety and depression as predictors vs mediators of paranoia.

Factors	Outcome	BIC	Beta	Std. Error	df	*t*	*p*	95% CI	Indirect effect (A*B)	Total effect (C)	Proportion mediated (%)
**Total effect (Path C)**											
t−1 insomnia	paranoia	95.3	1.826	0.59	50.0	3.11	0.003	0.6, 3.0			
t−1 anxiety	paranoia	94.4	0.748	0.20	48.2	3.74	0.001	0.3, 1.2			
t−1 depression	paranoia	91.4	0.839	0.20	45.8	4.20	<0.001	0.4, 1.2			
**Indirect effect (Path A)**											
t−1 insomnia	anxiety	27.4	1.511	0.30	48.32	5.04	<0.001	0.9, 2.1			
t−1 insomnia	depression	60.2	2.019	0.41	49.77	4.92	<0.001	1.2, 2.8			
t−1 anxiety	insomnia	−56.0	0.191	0.04	49.7	4.78	<0.001	0.1, 0.3			
t−1 depression	insomnia	−55.2	0.187	0.05	42.86	3.74	<0.001	0.1, 0.3			
**Mediation models (Path B, Path C’)**											
t−1 insomnia *(predictor)*	paranoia	66.8	−0.144	0.53	44.6	−0.27	0.788	−1.2, 0.9	2.079	1.826	113.9^a^
anxiety *(mediator)*			1.376	0.21	48.4	6.55	<0.001	1.0, 1.8			
t−1 insomnia *(predictor)*	paranoia	45.6	−0.461	0.43	48.2	−1.08	0.286	−1.3, 0.4	2.302	1.826	126.1^a^
depression *(mediator)*			1.140	0.12	48.5	9.49	<0.001	0.9, 1.4			
t−1 anxiety *(predictor)*	paranoia	86.1	0.361	0.22	48.5	1.63	0.109	−0.1, 0.8	0.349	0.748	46.7
insomnia *(mediator)*			1.826	0.59	48.5	3.07	0.004	0.6, 3.0			
t−1 depression *(predictor)*	paranoia	83.6	0.488	0.21	38.8	2.35	0.026	0.1, 0.9	0.311	0.839	37.1
insomnia *(mediator)*			1.663	0.58	48.8	2.89	0.006	0.5, 2.8			

t−1 = previous time point.

a Proportions over 100 indicate an independent relationship of the mediator on to the outcome, separate from its role in mediating the effect of the predictor.

Table 5 shows the results for hallucinations from this analysis. Insomnia, anxiety, and depression severity were all individually significant predictors of later hallucinations. However, as with paranoia, in the mediation models it was clear that the models with insomnia as predictor and negative affect as mediator (BICs of 70.2 and 79.5) provided a better fit to the observed data than the oppositional models (BICs of 91.6 and 88.4). The model comparison for insomnia and anxiety indicates ‘very strong’ support for a significant difference (BIC difference = 21.2), while the model comparison for insomnia and depression indicates `strong’ support (BIC difference = 8.9; Kass and Raftery, 1995) . Furthermore, a higher proportion of effect was mediated in the hypothesised models than in the oppositional models. The hypothesised models for paranoia mediated a higher proportion of the relationship than the hypothesised models for hallucinations, indicating that negative affect has a greater mediator role for insomnia leading to paranoia than insomnia and hallucinations.

**Table 5:** Insomnia, anxiety, and depression as predictors vs mediators of hallucinations.

Factors	Outcome	BIC	Beta	Std. Error	df	*t*	*p*	95% CI	Indirect effect (A*B)	Total effect (C)	Proportion mediated (%)
**Total effect (Path C)**											
t−1 insomnia	hallucinations	95.9	1.262	0.59	49.3	2.14	0.038	0.1, 2.1			
t−1 anxiety	hallucinations	96.6	0.446	0.21	48.8	2.13	0.035	0.0, 0.9			
t−1 depression	hallucinations	91.9	0.644	0.20	45.1	3.20	0.003	0.2, 1.0			
**Indirect effect (Path A)**											
t−1 insomnia	anxiety	27.4	1.511	0.30	48.32	5.04	<0.001	0.9, 2.1			
t−1 insomnia	depression	60.2	2.019	0.41	49.77	4.92	<0.001	1.2, 2.8			
t−1 anxiety	insomnia	−56.0	0.191	0.04	49.7	4.78	<0.001	0.1, 0.3			
t−1 depression	insomnia	−55.2	0.187	0.05	42.86	3.74	<0.001	0.1, 0.3			
**Mediation models (Path B, Path C’)**											
t−1 insomnia *(predictor)*	hallucinations	70.2	−0.954	0.56	45.9	−1.72	0.093	−2.1, 0.2	0.870	1.081	80.5
anxiety *(mediator)*			1.236	0.21	41.2	5.90	<0.001	0.8, 1.7			
t−1 insomnia *(predictor)*	hallucinations	79.5	−0.546	0.59	43.1	−0.93	0.347	−1.7, 0.7	0.779	1.081	72.1
depression *(mediator)*			0.777	0.17	43.9	4.59	<0.001	0.4, 1.1			
t−1 anxiety *(predictor)*	hallucinations	91.6	0.210	0.23	48.9	0.90	0.374	−0.3, 0.7	0.230	0.466	49.4
insomnia *(mediator)*			1.309	0.63	49.0	2.08	0.043	0.0, 2.6			
t−1 depression *(predictor)*	hallucinations	88.4	0.462	0.23	41.8	2.02	0.048	0.0, 0.9	0.186	0.644	28.9
insomnia *(mediator)*			0.997	0.61	49.0	1.65	0.106	−0.2, 2.2			

t−1 = previous time point.

In summary, insomnia was a significant predictor of later paranoia and later hallucinations, supporting our second hypothesis. The third hypothesis was also supported; models where insomnia was the predictor and negative affect was the mediator were a better fit than oppositional models where these roles were reversed.

### 3.4 Phase three: comparing direction of effects

Table 6 contains results comparing the prediction of insomnia by paranoia versus prediction of paranoia by insomnia. Both insomnia and paranoia significantly predicted each other over time. However, the *t*-statistics between Models 1, 2, and 4 for each direction did show differences. For Model 1 (cross-sectional), the insomnia to paranoia direction had a higher *t*-value than the reverse direction (5.06 compared to 4.23), indicating this has the highest effect. Moving to Model 2, which tested longitudinal prediction, the direction from paranoia to insomnia instead had the highest *t*-statistic by a small margin (3.53 versus 3.09 for the hypothesised model). For Model 4, where the model was adjusted for the previous level of the outcome, the oppositional model again had a higher *t* statistic, although again the margin was small (1.07, versus 0.97 for the hypothesised model). This supports a bidirectional relationship between insomnia and paranoia, as the differences in *t*-statistics between the hypothesised and oppositional models are not large enough to be significant (all differences are less than 1, which is the standard deviation of the t distribution).

**Table 6:** Direction analysis comparison between insomnia and paranoia.

	Factor	Outcome	BIC	Beta	Std. Error	df	*t*	*p*	95% CI
**Insomnia to paranoia**									
Model 1	insomnia	paranoia	138.0	2.073	0.41	80.1	5.06	<0.001	1.3, 2.9
Model 2	t−1 insomnia	paranoia	95.3	1.826	0.59	50.0	3.09	0.003	0.6, 3.0
Model 3	t−1 paranoia	paranoia	69.28	0.884	0.11	45.4	8.04	<0.001	0.7, 1.1
Model 4	t−1 insomnia	paranoia	68.07	0.448	0.46	41.1	0.97	0.336	−0.5, 1.3
	t−1 paranoia			0.829	0.12	45.2	6.91	<0.001	0.6, 1.1
**Paranoia to insomnia**									
Model 1	paranoia	insomnia	−57.3	0.127	0.03	46.8	4.23	<0.001	0.1, 0.2
Model 2	t−1 paranoia	insomnia	−48.8	0.106	0.03	48.8	3.53	0.001	0.0, 0.2
Model 3	t−1 insomnia	insomnia	−84.7	0.789	0.10	49.5	7.89	<0.001	0.6, 1.0
Model 4	t−1 paranoia	insomnia	−80.8	0.032	0.03	48.9	1.07	0.290	0.0, 0.1
	t−1 insomnia			0.739	0.10	48.5	7.39	<0.001	0.5, 0.9

t−1 = previous time point.

Table 7 contains the results from the same analysis for insomnia and hallucinations. Cross-sectionally (Model 1) hallucinations were slightly more predictive of insomnia than vice versa (*t *= 2.94 vs *t *= 3.23). However, in both longitudinal analyses (Models 2 and 4) the hypothesised models (*t *= 2.14, *t *= 1.25) with insomnia as prior show a stronger effect than the oppositional models (*t *= 1.68, *t *= 1.00). As with paranoia, these differences in *t* statistics are not large enough to be considered significant. However, the uncorrected oppositional model (Model 2) indicated that previous hallucinations was itself not a significant predictor (*p *= 0.085) for later insomnia in this study. Overall these results therefore support that insomnia was a stronger predictor of hallucinations than vice versa.

**Table 7:** Directional analysis comparison between insomnia and hallucinations.

	Factor	Outcome	BIC	Beta	Std. Error	df	*t*	*p*	95% CI
**Insomnia to Hallucinations**									
Model 1	insomnia	hallucinations	91.3	1.589	0.54	49.5	2.94	0.005	0.5, 2.7
Model 2	t−1 insomnia	hallucinations	95.9	1.262	0.59	49.3	2.14	0.038	0.1, 2.1
Model 3	t−1 hallucinations	hallucinations	59.6	0.833	0.10	43.8	8.33	<0.001	0.6, 1.0
Model 4	t−1 insomnia	hallucinations	58.2	0.498	0.40	45.8	1.25	0.218	−0.3, 1.3
	t−1 hallucinations			0.820	0.10	44.1	8.20	<0.001	0.6, 1.0
**Hallucinations to Insomnia**									
Model 1	hallucinations	insomnia	−48.8	0.097	0.03	45.4	3.23	0.002	0.0, 0.2
Model 2	t−1 hallucinations	insomnia	−32.1	0.067	0.04	50.0	1.68	0.099	0.0, 0.1
Model 3	t−1 insomnia	insomnia	−84.7	0.789	0.10	49.5	7.89	<0.001	0.6, 1.0
Model 4	t−1 hallucinations	insomnia	−80.6	0.030	0.03	47.5	1.00	0.322	0.0, 0.1
	t−1 insomnia			0.769	0.10	48.4	7.69	<0.001	0.6, 1.0

t−1 = previous time point.

In summary the fourth hypothesis regarding a role of psychotic experiences in predicting insomnia was partially supported: paranoia significantly predicted later insomnia, but hallucinations did not clearly predict later insomnia in our results. The final hypothesis – that the insomnia to psychotic experience relationship would be stronger than the reverse direction – was also partially supported. The insomnia to hallucinations relationship appeared stronger than the reverse direction, but the relationship between insomnia and paranoia was bidirectional.

## 4 Discussion

This was the first longitudinal study to investigate clinical trends in insomnia and psychotic symptoms over a number of months in patients with early non-affective psychosis. The results support the key hypotheses that insomnia is a significant predictor of paranoia and hallucinations both within and across time, with the relationships mediated by negative affect (depression and anxiety). This is especially the case for paranoia, where negative affect completely mediated the effect of insomnia on paranoia, whereas only partial mediation was demonstrated for hallucinations. A novel finding is that paranoia was also a significant predictor of later insomnia, supporting a conceptualization of a bidirectional relationship. However, the relationship between insomnia and hallucinations appears to move more strongly in one direction – from insomnia to hallucinations – than in the reverse direction. These findings clearly endorse the importance of insomnia as a factor in the maintenance of paranoia and hallucinations, while also providing further detail on the interaction between these symptoms.

These findings support a role for negative affect as mediating the relationship between insomnia and psychotic experiences as found in a recent non-clinical manipulation study (Reeve et al., 2018). This is also consistent with well-known links between insomnia and negative affect, and also with the importance of affective processes in theoretical models of psychotic experiences (Freeman and Garety, 2003). It is interesting that negative affect mediated a larger proportion of the relationship between insomnia and paranoia than insomnia and hallucinations. This has been reported elsewhere (Reeve et al., 2018), and is consistent with a larger evidence base for the role of anxiety and depression in paranoia than in hallucinations (Hartley et al., 2013). Furthermore, the results here indicate that treatment of insomnia would be likely to improve affective symptoms, which besides their role in psychotic experiences, are additionally associated with significant distress and disability (Koyanagi et al., 2017, Koyanagi et al., 2016).

The results of this study have clinical implications regarding the importance of treating insomnia in people with psychosis, even more so given that the influence of psychotic experiences on insomnia was taken into account. The finding that paranoia also increases insomnia highlights the importance of targeting both paranoia and insomnia in this group – if treating insomnia improves paranoia, and paranoia improves insomnia, it is possible that treatment for one factor might instil a virtuous cycle of symptom improvement for both issues. For hallucinations, the finding that insomnia severity predicts later hallucination severity but not vice versa supports a potential role for insomnia treatment in improvement of hallucination severity. The results therefore support a role of treating insomnia to improve psychotic experiences (Freeman et al., 2017).

A recent survey found that clinicians rarely utilise formal assessments or recommended interventions for sleep disorders (Rehman et al., 2016). Yet the feasibility, acceptability, and effectiveness of cognitive behavioural therapy for insomnia, with appropriate adaptations, has been demonstrated for patients with persistent psychosis (Freeman et al., 2015, Waite et al., 2016b), individuals at-risk of psychosis (Bradley et al., 2018), and inpatients (Sheaves et al., 2017). In all these studies, improvements in insomnia were large (d ≥ 0.9), and uptake of treatment was high (96% across the three trials listed). As discussed earlier, cognitive behavioural therapy for insomnia has also been shown to improve non-clinical psychotic experiences, and negative affect, in students with insomnia (Freeman et al., 2017). These recent advances, alongside the current study, indicate that the treatment of insomnia should be given a higher priority in mental health services.

### 4.1 Limitations and conclusion

One limitation is that it is not possible to tell if the study group is representative of the participant population in general – it may be that those with insomnia were more likely to take part in the study and attend follow up appointments, potentially inflating the relationships reported here. However, this potential bias was minimised wherever possible by pro-active approaches for follow-up and flexibility in assessments, as demonstrated by the high retention rate within the study. It is also worth noting that all participants in the study were currently receiving care within mental health services (with the majority prescribed antipsychotic medication).

A generic limitation of longitudinal observational studies is the assumption that priority is suggestive of a causal role, as it could be the case that insomnia (or anxiety and depression) might be more readily acknowledged by the participants than psychotic experience. In this case evidence of prior occurrence could be an artefact of a lower threshold for awareness of the issue. However, in this study all outcomes were assessed at each time point using the same questionnaires, therefore it is difficult to see how the threshold for acknowledging symptoms would change over the course of the study, especially when the general trend was for improvement in symptoms. Another general issue with longitudinal observational studies is that a common cause cannot be ruled out as the key explanatory factor.

In conclusion, this study provides evidence that insomnia symptoms are predictive of changes in psychotic experiences in an early psychosis clinical group, with this relationship strongly mediated by insomnia predicting later negative affect. Importantly, this study is the first to indicate that the predictive relationship from insomnia to psychotic experiences has the same or greater strength than the reverse relationship in a clinical group, prompting a rethink of traditional conceptualisations of insomnia as a secondary concern in psychosis. Furthermore, given the existence of an effective insomnia intervention for this group (CBTi; Freeman et al., 2015), these results strongly support further research investigating if treating insomnia improves clinical trajectory for individuals with early psychosis.

## References

[bib0001] Baron R.M., Kenny D.A (1986). The moderator-mediator variable distinction in social psychological research: conceptual, strategic, and statistical considerations. J. Pers. Soc. Psychol..

[bib0002] Benoit K. (2011). Linear Regression Models With Logarithmic Transformations.

[bib0003] Box G.E.P., Cox D.R., Society S., Methodological S.B. (1964). An analysis of transformations. Analysis.

[bib0004] Bradley J., Freeman D., Chadwick E., Harvey A.G., Mullins B., Johns L. (2018). Treating sleep problems in young people at ultra-high risk of psychosis: a feasibility case series. Behav. Cogn. Psychother..

[bib0005] Chiu V.W., Ree M., Janca A., Waters F. (2016). Sleep in schizophrenia: exploring subjective experiences of sleep problems, and implications for treatment. Psychiatr. Q..

[bib0006] Christensen H., Batterham P.J., Gosling J.A., Ritterband L.M., Griffiths K.M., Thorndike F.P. (2016). Effectiveness of an online insomnia program (SHUTi) for prevention of depressive episodes (the GoodNight Study): a randomised controlled trial. Lancet Psychiatry.

[bib0007] Davies G., Haddock G., Yung A., Mulligan L.D., Kyle S. (2017). A systematic review of the nature and correlates of sleep disturbance in early psychosis. Sleep Med. Rev..

[bib0008] Espie C. (1991). The Psychological Treatment of Insomnia.

[bib0009] Freeman D., Garety P. (2003). Connecting neurosis and psychosis: the direct influence of emotion on delusions and hallucinations. Behav. Res. Ther..

[bib0010] Freeman D., Pugh K., Vorontsova N., Southgate L. (2009). Insomnia and paranoia. Schizophr. Res..

[bib0011] Freeman D., Sheaves B., Goodwin G.M., Yu L.M., Nickless A., Harrison P.J. (2017). The effects of improving sleep on mental health (OASIS): a randomised controlled trial with mediation analysis. Lancet Psychiatry.

[bib0012] Freeman D., Waite F., Startup H., Myers E., Lister R., McInerney J. (2015). Efficacy of cognitive behavioural therapy for sleep improvement in patients with persistent delusions and hallucinations (BEST): a prospective, assessor-blind, randomised controlled pilot trial. Lancet Psychiatry.

[bib0013] Hartley S., Barrowclough C., Haddock G. (2013). Anxiety and depression in psychosis: a systematic review of associations with positive psychotic symptoms. Acta Psychiatr. Scand..

[bib0014] Harvey A. (2002). A cognitive model of insomnia. Behav. Res. Ther.

[bib0015] Harvey A., Murray G. (2011). Sleep disturbance as transdiagnostic: consideration of neurobiological mechanisms. Clin. Psychol. Rev..

[bib0016] Hennig T., Lincoln T.M. (2018). Sleeping paranoia away? An actigraphy and experience-sampling study with adolescents. Child Psychiatry Hum. Dev..

[bib0017] Hodgekins J., French P., Birchwood M., Mugford M., Christopher R., Marshall M. (2015). Comparing time use in individuals at different stages of psychosis and a non-clinical comparison group. Schizophr. Res..

[bib0018] IBM Corp. (2015). IBM SPSS Statistics.

[bib0019] Jansson-Fröjmark M., Lindblom K. (2008). A bidirectional relationship between anxiety and depression, and insomnia? A prospective study in the general population. J. Psychosom. Res..

[bib0020] Kass R.E., Raftery A.E. (1995). Bayes factors. J. Am. Stat. Assoc.

[bib0021] Keene O.N. (1995). The log trasnformation is special. Stat. Med..

[bib0022] Koyanagi A., Oh H., Stickley A., Haro J.M., DeVylder J. (2016). Risk and functional significance of psychotic experiences among individuals with depression in 44 low- and middle-income countries. Psychol. Med..

[bib0023] Koyanagi A., Oh H., Stubbs B., Haro J.M., DeVylder J.E. (2017). Epidemiology of depression with psychotic experiences and its association with chronic physical conditions in 47 low- and middle-income countries. Psychol. Med..

[bib0024] Koyanagi A., Stickley A. (2015). The association between sleep problems and psychotic symptoms in the general population: a global perspective. Sleep.

[bib0025] Li L., Wu C., Gan Y., Qu X., Lu Z. (2016). Insomnia and the risk of depression: a meta-analysis of prospective cohort studies. BMC Psychiatry.

[bib0026] Lovibond S.H., Lovibond P.F. (1995). Manual for the Depression Anxiety Stress Scales.

[bib0027] Mulligan L.D., Haddock G., Emsley R., Neil S.T., Kyle S. (2016). High resolution examination of the role of sleep disturbance in predicting functioning and psychotic symptoms in schizophrenia: a novel experience sampling study. J. Abnorm. Psychol..

[bib0028] Neckelmann D., Mykletun A., Dahl A.A. (2007). Chronic insomnia as a risk factor for developing anxiety and depression. Sleep.

[bib0029] Reeve S., Emsley R., Sheaves B., Freeman D. (2018). Disrupting sleep: the effects of sleep loss on psychotic experiences tested in an experimental study with mediation analysis. Schizophr. Bull..

[bib0030] Reeve S., Sheaves B., Freeman D. (2015). The role of sleep dysfunction in the occurrence of delusions and hallucinations: a systematic review. Clin. Psychol. Rev..

[bib0031] Rehman A., Waite F., Sheaves B., Biello S., Freeman D., Gumley A. (2016). Clinician perceptions of sleep problems, and their treatment, in patients with non-affective psychosis. Psychosis.

[bib0032] Ronald A., Sieradzka D., Cardno A.G., Haworth C.M., McGuire P., Freeman D. (2014). Characterization of psychotic experiences in adolescence using the specific psychotic experiences questionnaire: findings from a study of 5000 16-Year-Old Twins. Schizophr. Bull..

[bib0033] Sheaves B., Freeman D., Isham L., McInerney J., Nickless A., Yu L.-M. (2017). Stabilising sleep for patients admitted at acute crisis to a psychiatric hospital (OWLS): an assessor-blind pilot randomised controlled trial. Psychol. Med..

[bib0034] Spoormaker V.I., Verbeek I., van den Bout J., Klip E.C. (2005). Initial validation of the SLEEP-50 questionnaire. Behav. Sleep Med..

[bib0035] Stubbs B., Williams J., Gaughran F., Craig T. (2016). How sedentary are people with psychosis? A systematic review and meta-analysis. Schizophr. Res..

[bib0036] Waite F., Evans N., Myers E., Startup H., Lister R., Harvey A. (2016). The patient experience of sleep problems and their treatment in the context of current delusions and hallucinations. Psychol. Psychother. Theory Res. Pract..

[bib0037] Waite F., Myers E., Harvey A., Espie C., Startup H., Sheaves B. (2016). Treating sleep problems in patients with schizophrenia. Behav. Cogn. Psychother..

[bib0038] Waters F., Sinclair C., Rock D., Jablensky A., Foster R., Wulff K. (2011). Daily variations in sleep-wake patterns and severity of psychopathology: a pilot study in community-dwelling individuals with chronic schizophrenia. Psychiatry Res.

[bib0039] Xiang Y.-T., Weng Y.-Z., Leung C.-M., Tang W.-K., Lai K.Y.C., Ungvari G.S. (2009). Prevalence and correlates of insomnia and its impact on quality of life in Chinese schizophrenia patients. Sleep.

[bib3032] Zavos H.M.S., Freeman D., Haworth C.M.A., McGuire P., Plomin R., Cardno A.G. (2014). Consistent etiology of severe, frequent psychotic experiences and milder, less frequent manifestations: A twin study of specific psychotic experiences in adolescence. JAMA Psychiatry.

